# Supplementary material for: A morpheein equilibrium regulates catalysis in phosphoserine phosphatase SerB2 from Mycobacterium tuberculosis
Source: Commun Biol. 2023 Oct 10;6:1024. doi: 10.1038/s42003-023-05402-z (PMC10564941; doi:10.1038/s42003-023-05402-z)
Supplement: Supplementary file 2 — Supplementary information [file 42003_2023_5402_MOESM2_ESM.pdf]

Supplementary information

A morpheein equilibrium regulates catalysis in phosphoserine phosphatase SerB2 from *Mycobacterium tuberculosis*

Elise Pierson[1], Florian De Pol[1], Marianne Fillet[2], and Johan Wouters[1,*]

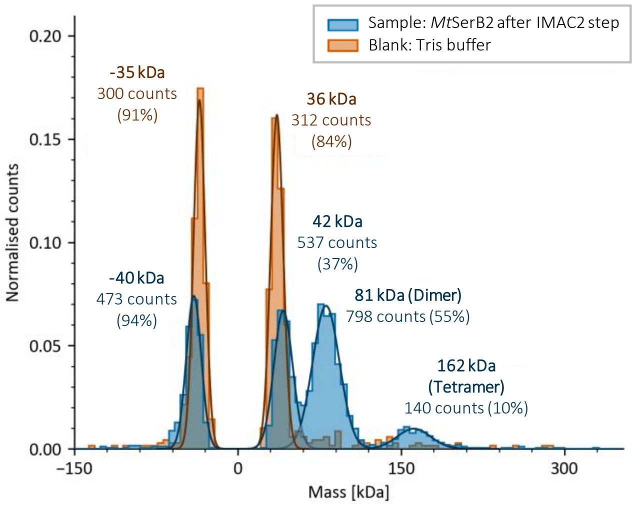

Supplementary Fig. 1. Mass photometry (MP) measurement on an unseparated *Mt*SerB2 sample. Superimposition of MP histograms of a *Mt*SerB2 sample analysed after the second step of purification by immobilized metal affinity chromatography (IMAC) and of only the Tris buffer in which the sample is diluted. Monomeric species in *Mt*SerB2 sample cannot be quantified due to the Tris buffer signal. According to the experiment, the dimer/tetramer count ratio is about 85/15 (798/140).

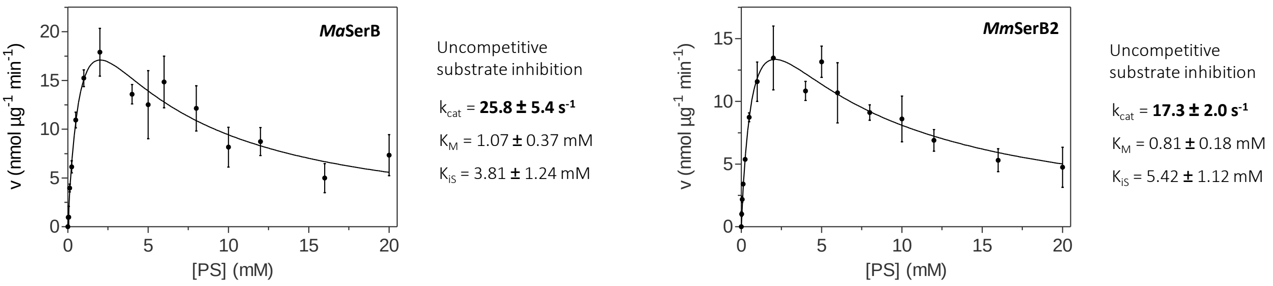

Supplementary Fig. 2 Evaluation of the kinetics of phosphoserine (PS) dephosphorylation by *Ma*SerB dimer and *Mm*SerB2 dimer. Plots of initial velocity (nmol phosphate released per µg of enzyme per minute) versus substrate concentration for PS dephosphorylation by *Ma*SerB dimer (left panel) or *Mm*SerB2 dimer (right panel). Error bars represent the s.d. of three experiments. The corresponding kinetic parameters were calculated by fitting equation (1) describing total uncompetitive substrate inhibition and are shown next to the plots. Source data are available on FigShare repository (https://doi.org/10.6084/ m9.figshare.24116571).

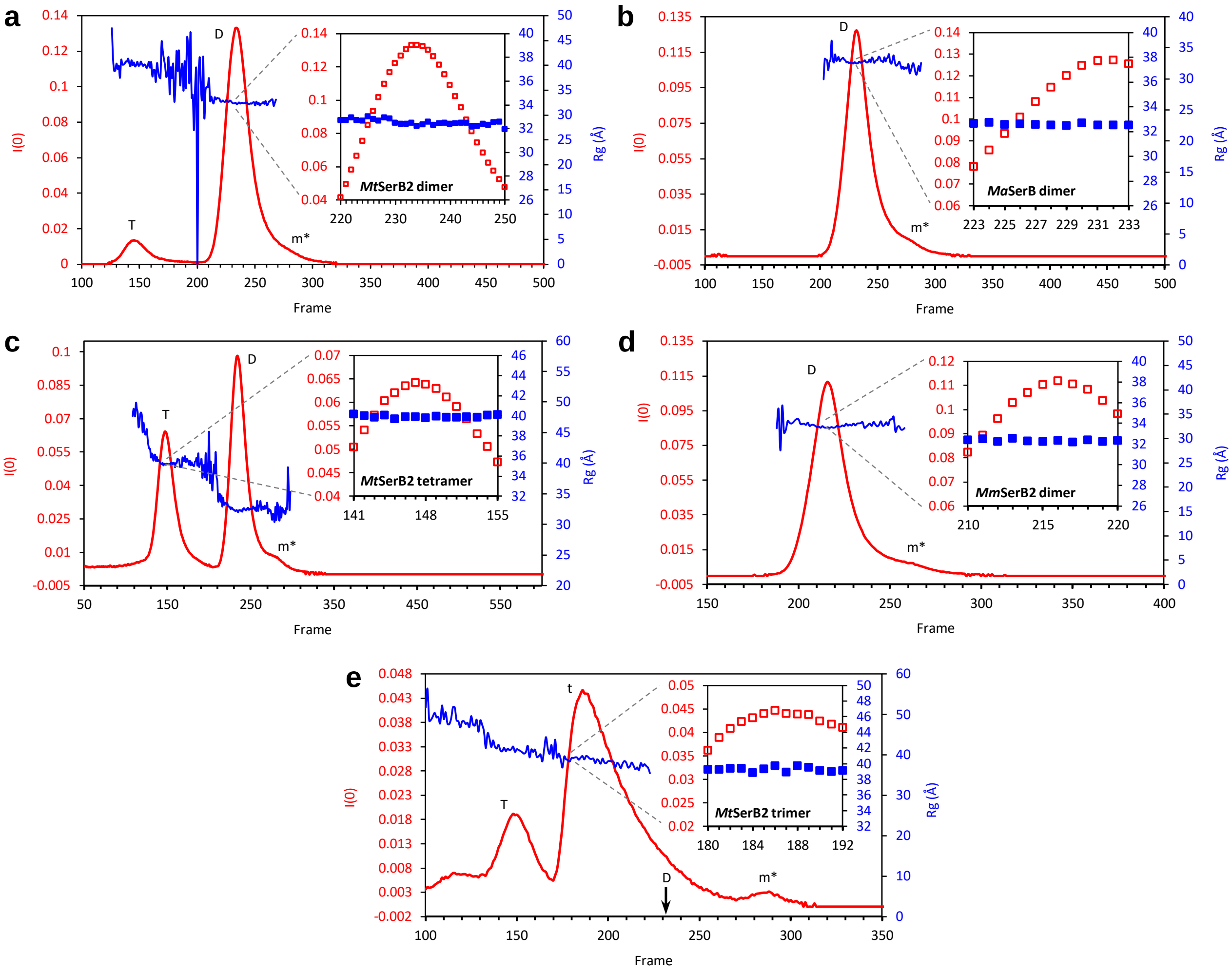

Supplementary Fig. 3 Assessment of the monodispersity of the oligomeric species and measurement frame selection during SEC-UV-SAXS experiments. Plots of I(0) and R_g as a function of measurement frame (1 second) for SEC-UV-SAXS analyses of (**a**) *Mt*SerB2 dimer fraction pool, (**b**) *Ma*SerB, (**c**) *Mt*SerB2 after the second step of purification by immobilized metal affinity chromatography (reverse IMAC), (**d**) *Mm*SerB2, and (**e**) *Mt*SerB2 after reverse IMAC in the presence of L-serine. Analyses **a-d** were performed in A2 buffer (50 mM Tris-HCl pH 7.4, 150 mM NaCl, 1 mM TCEP) and analysis **e** in A2Ser buffer (A2 buffer, 10 mM L-Ser) on a BioResolveSEC mAb 200 Å 2.5 µm 7.8 x 300 mm column (*Waters*) preceded by a BioResolve Sec mAb 200 Å 2.5 µm 4.6 x 30 mm 229 precolumn (*Waters*). Inserts are close-ups on the data frames selected for averaging (similar R_g within 0.2 (**a-d**) or 0.3 Å (**e**) error) to obtain I(q) *versus* q curves for (**a**) *Mt*SerB2 dimer, (**b**) *Ma*SerB dimer, (**c**) *Mt*SerB2 tetramer, (**d**) *Mm*SerB2 dimer and (**e**) *Mt*SerB2 trimer. T = tetramer, D = dimer, t = trimer, m* = probable monomer. The arrow in chromatogram **e** indicates the retention time of dimer peak of chromatogram **c** for assessing the peak displacement induced by L-Ser.

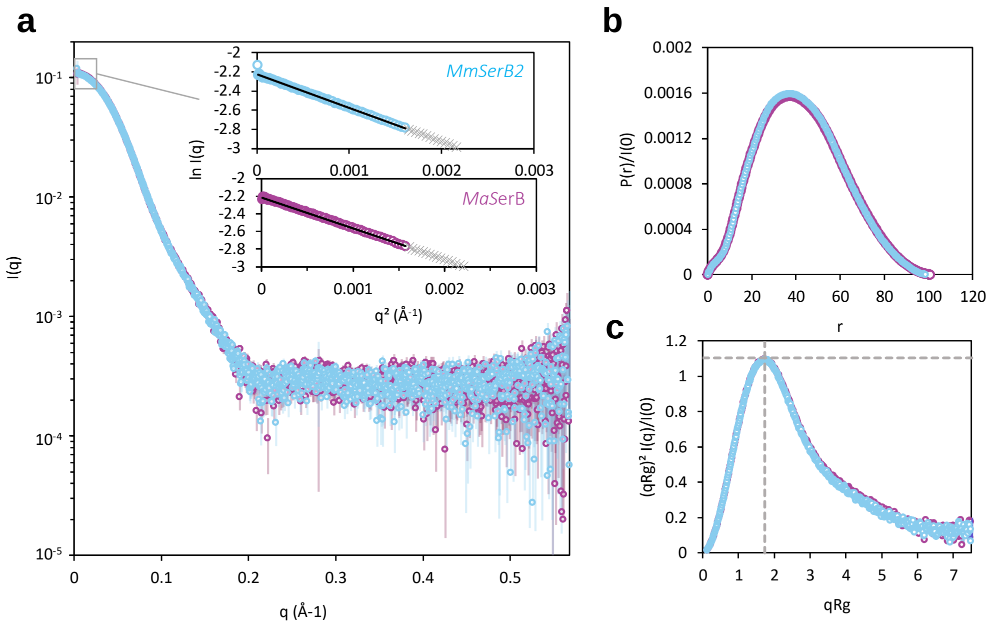

Supplementary Fig. 4 Comparison of the SAXS solution structures of *Mm*SerB2 and *Ma*SerB dimers. a Superimposition of *I(q)* versus q as log-linear plots for *Mm*SerB2 and *Ma*SerB dimers. The inset shows the Guinier fit (coloured symbols) for qR_g < 1.3 with cross symbols (grey) indicating data beyond the Guinier region. **b** Dimensionless Kratky plots for the data in panel **a**. **c** *P(r)* functions from the data in panel **a** normalized to *I(0)* for comparison purposes.

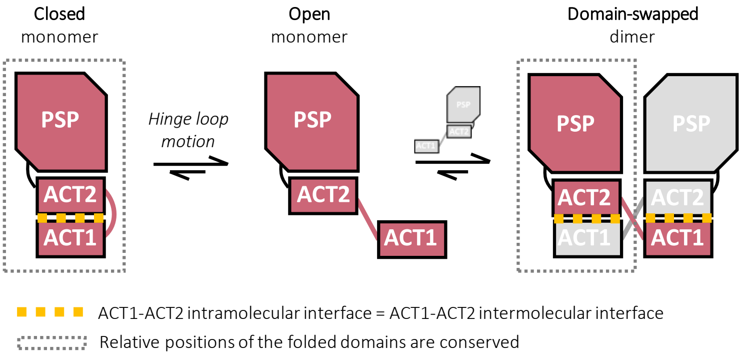

Supplementary Fig. 5 Schematic representation of the formation of *Mt*SerB2 domain-swapped dimer. The two monomers that associate are depicted in grey and red, respectively. Only the opening via hinge-loop (red line) motion of the red monomer is shown. Interactions between the ACT1 and ACT2 domains are represented by yellow dashes.

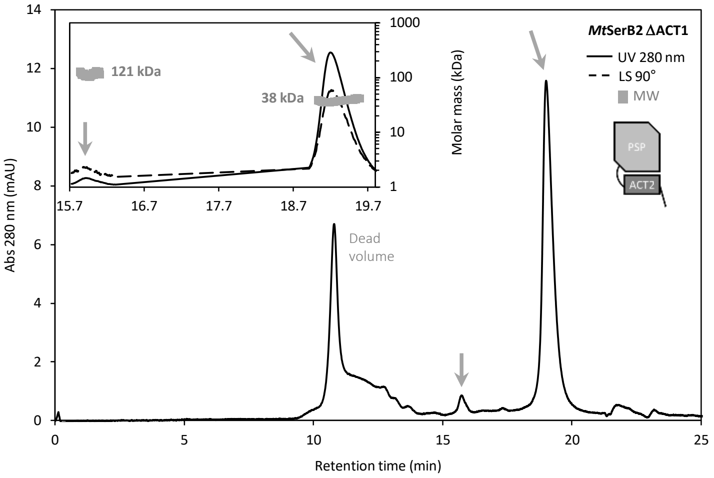

Supplementary Fig. 6 Analysis of the oligomeric state of truncation variant *Mt*SerB2ΔACT1 (35 kDa) by SEC-UV-MALS. The main chromatogram shows the elution monitored by absorbance at 280 nm and the inset gives a close-up on this signal, superimposed on the light scattering signal measured at 90°. *Mt*SerB2ΔACT1 elutes in a majority peak corresponding to a measured molar mass of 38 kDa (monomer). The small peak eluting before the majority peak and the aggregates that exit in the column dead volume corresponds to a measured molar mass of 121 kDa which could indicate the presence of a trimeric population (121/38 ≈ 3.2).

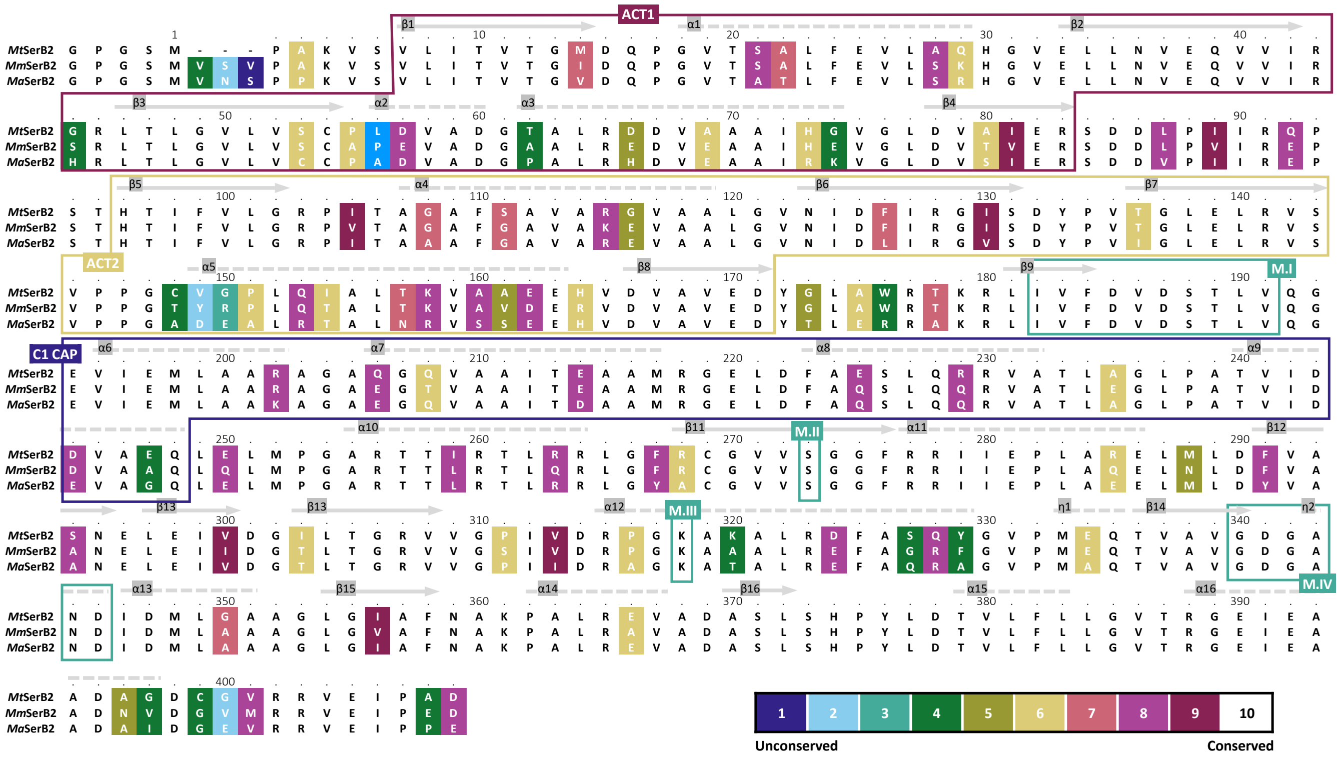

Supplementary Fig. 7 Multiple sequence alignment of *M. tuberculosis* SerB2 (*Mt*SerB2), *M. marinum* SerB2 (*Mm*SerB2), and *M. avium* SerB (*Ma*SerB). The alignment was performed with the program *PRALINE* (www.ibi.vu.nl/programs/pralinewww/). The secondary structure elements of *Ma*SerB (PDB: 3P96) are shown in the first line. The residues are coloured according to their degree of conservation between the three sequences according to the legend at the bottom right. Key regions are boxed: the ACT1 (purple) and ACT2 (yellow) domains, the C1 cap module (dark blue) and haloacid dehalogenase phosphatases key motifs I (hhhDxDx[T/V][L/V][L/I/V]h, II [S/T], III and IV ($K_{18\text{-}30}$[G/S][D/S]$x_{3\text{-}4}$[D/E]) where h is an hydrophobic residue and x is any residue.

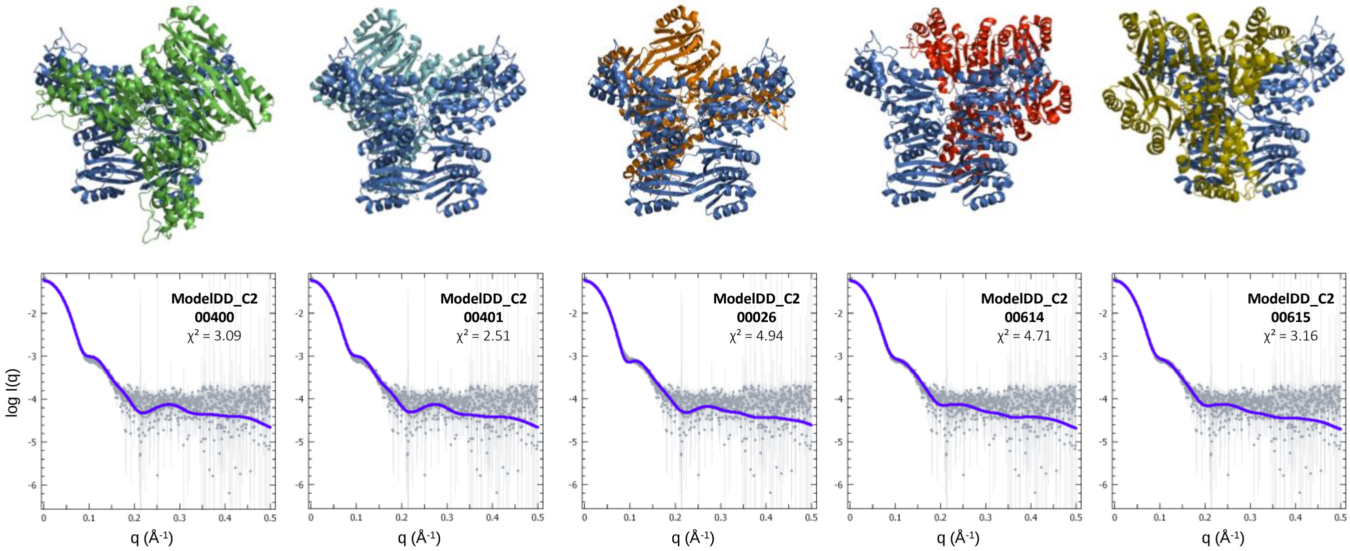

Supplementary Fig. 8 Dimer-of-dimers models fitting the experimental SAXS data of *Mt*SerB2 tetramer with χ^2 values below 5.00. The models were obtained through a C2 symmetric protein-protein docking of *Mt*SerB2 dimer homology model. Cartoon representations of the models are shown in the above panel and the corresponding fits to the experimental SAXS data with *CRYSOL* along their χ^2 values are shown in the bottom panel.

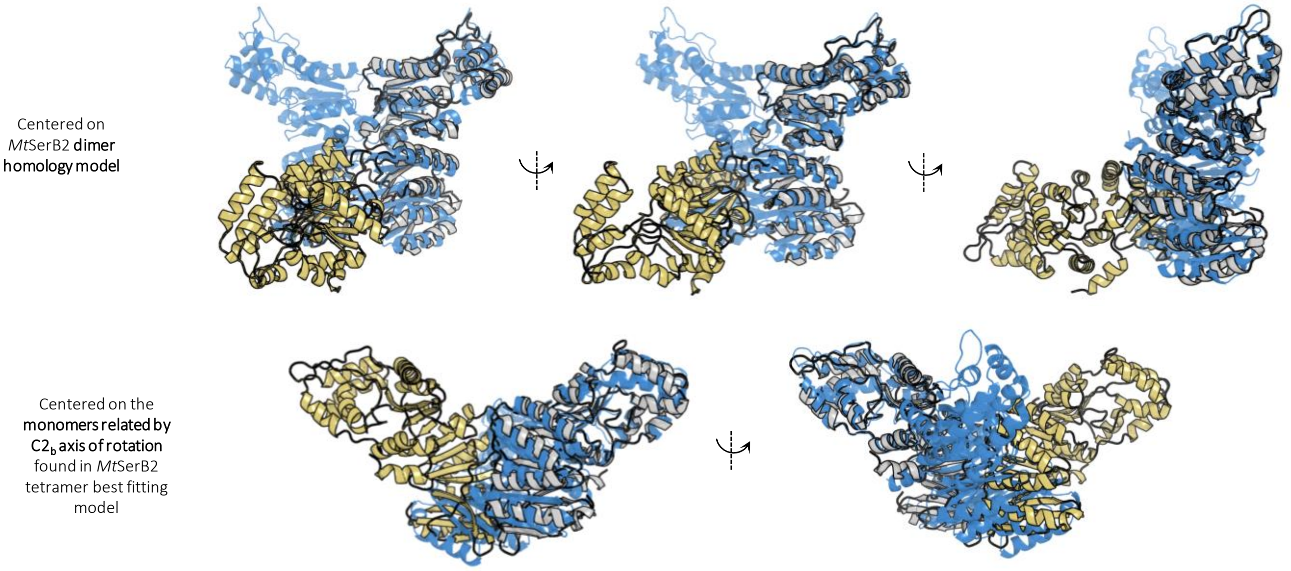

Supplementary Fig. 9 Superimposition of the structures of *Mt*SerB2 dimer homology model (blue) and of the monomers related by $C2_b$ axis of rotation (yellow and grey) found in *Mt*SerB2 tetramer model that best fits the SAXS data. Only one couple of monomers (yellow and grey) is depicted but the above figure can also be reproduced with the green-pink couple (see main text). The various orientations depicted show that the global architecture of the couple of $C2_b$ related monomers found in *Mt*SerB2 tetramer model is distinct from the architecture of *Mt*SerB2 domain-swapped dimer homology model.

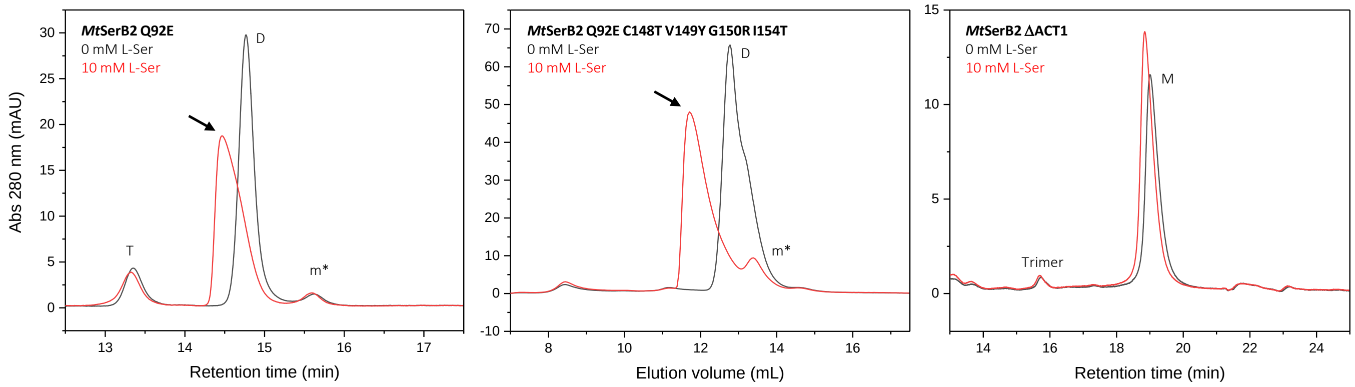

Supplementary Fig. 10 Study of the oligomeric behaviour of *Mt*SerB2 variants in the presence and absence of L-Ser by size exclusion chromatography. Superimposition of the chromatograms obtained during the SEC-UV analysis of *Mt*SerB2 variants impaired in their tetramerization ability: Q92E (left panel) and Q92E C148T V149Y G150R I154T (middle panel), and of *Mt*SerB2 variant whose ACT1 domain has been truncated: *Mt*SerB2 ΔACT1 (right panel) in the absence (black) and presence of 10 mM L-Ser (red) in the mobile phase. The arrows highlight an L-Ser induced oligomeric transition (leftward dimer peak shift). T = tetramer, D = dimer, m* = monomer. Oligomeric stoichiometries have been assigned to the peaks using multi angle light scattering.

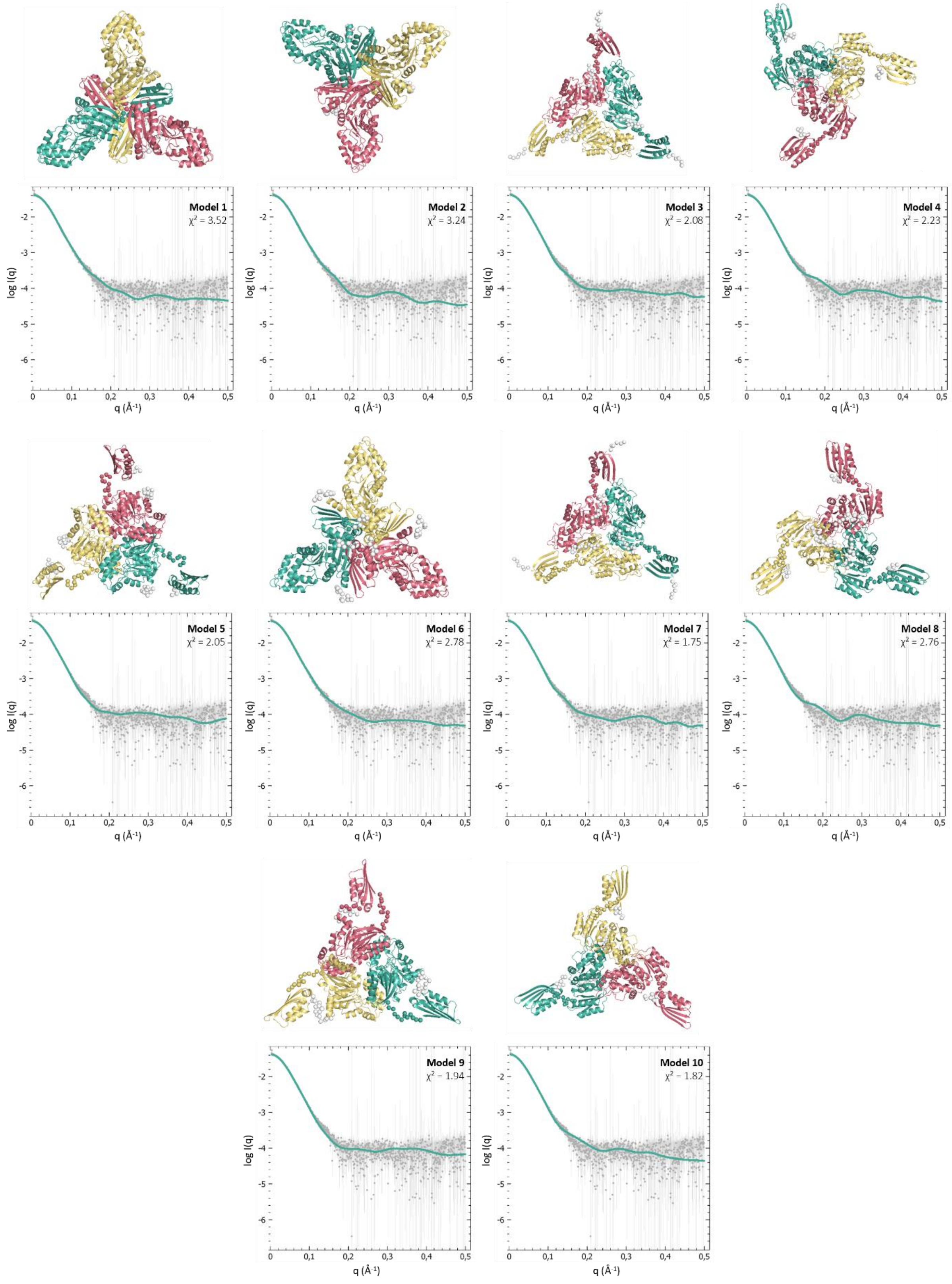

Supplementary Fig. 11 SAXS-based rigid body modelling of *Mt*SerB2 trimer. Cartoon representation of the three-dimensional structure of the ten models of *Mt*SerB2 trimers generated by SAXS-based rigid body modelling using *CORAL*. Flexible residues at the N- and C-terminal extremities (white) as well as the hinge loop (coloured) are represented as plain spheres. Below each model is shown the corresponding fit (green) to the experimental data as log *I(q)* versus q plots and χ^2 values calculated using *CRYSOL*.

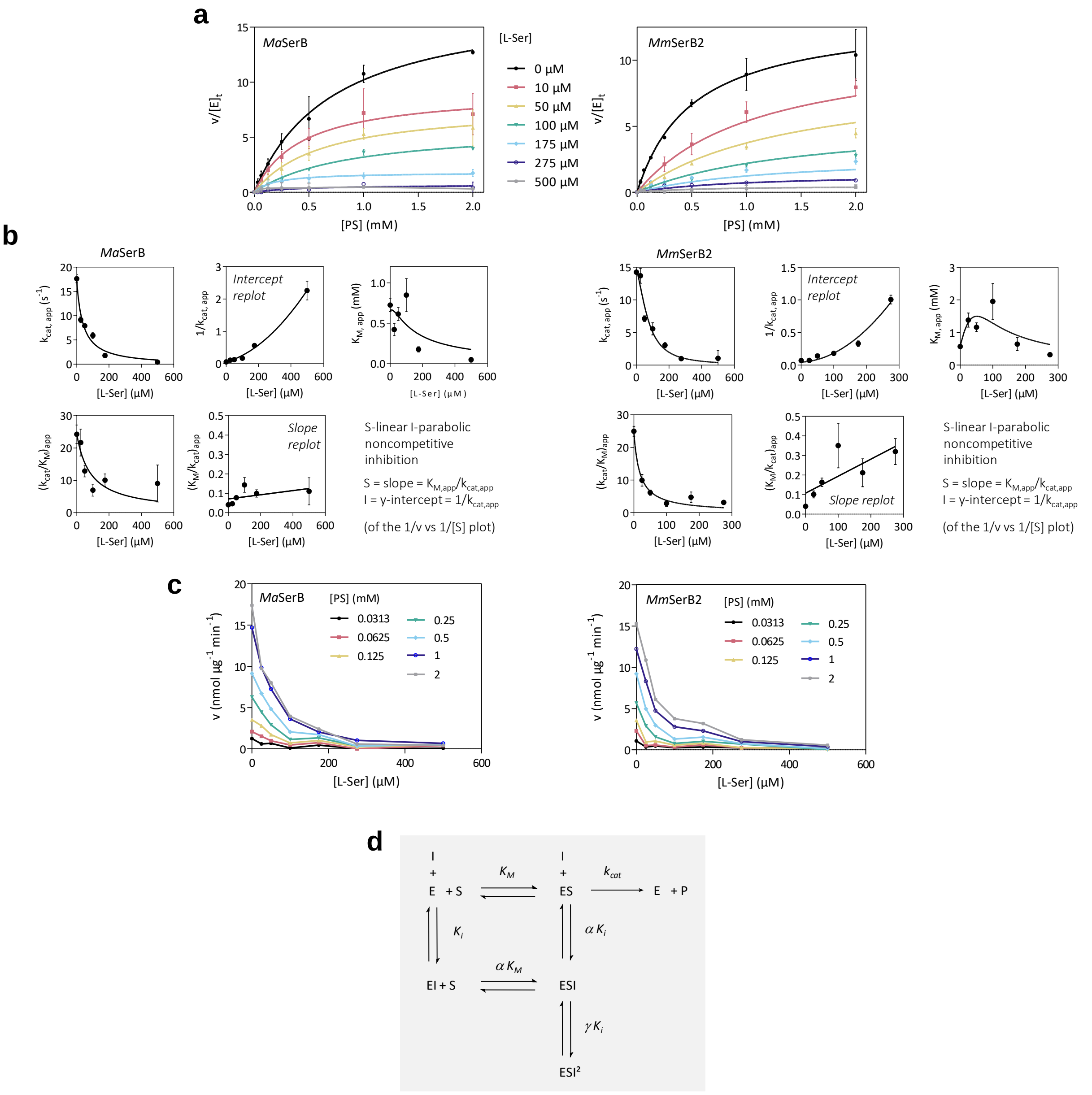

Supplementary Fig. 12 Evaluation of the effect of L-serine as a modifier of the kinetics of phosphoserine (PS) dephosphorylation by *Ma*SerB and *Mm*SerB2 dimers. a Plots of initial velocities (nmol phosphate released per µg of enzyme per minute) as $v/[E]_t$ (min^{-1}) versus substrate concentration for PS dephosphorylation by *Ma*SerB dimer (left) and *Mm*SerB2 dimer (right) at various fixed L-Ser concentrations (µM). Error bars represent the s.d. of three experiments. Plain lines are the fit of the general rate expression of S-linear I-parabolic noncompetitive inhibition (equation (8)) to the experimental data. Source data are available on FigShare repository (https://doi.org/10.6084/ m9.figshare.24116571). **b** Dependence of the apparent kinetic parameters kcat, K_M, (k_{cat}/K_M) on L-Ser concentration. The shape of the plots (especially the parabolic shape of $1/k_{cat,app}$ vs [L-Ser]) allowed the identification of a mechanism of S-linear I-parabolic noncompetitive inhibition. This type of inhibition involves the binding of two inhibitor molecules to the enzyme-substrate complex (see panel d). Plain lines are the fits of the dependency of apparent kinetic parameters on [L-Ser] to the experimental data (equation (9-13)) based on equation (8). Error bars represent the standard error of the parameter as calculated by Prism following non-linear regression. Source data are available on FigShare repository (https://doi.org/10.6084/ m9.figshare.24116571). **c** Plot of initial velocity versus L-Ser concentration at fixed PS concentrations. The velocity at high L-Ser concentration tends to zero and indicates a total ("linear") inhibition. **d** S-linear I-parabolic noncompetitive inhibition mechanism. E= enzyme, S = substrate (phosphoserine), P = product (phosphate), I = inhibitor (L-Ser), k_{cat} = catalytic constant, K_M = substrate dissociation constant, K_i = inhibition constant, α = reciprocal allosteric coupling constants between the substrate and the first inhibitor

molecule, γ = allosteric constant for the binding of the second inhibitor molecule, β = factor by which the modifier affects k_{cat}. Two inhibitor molecules bind to the enzyme-substrate complex (ESI^2).

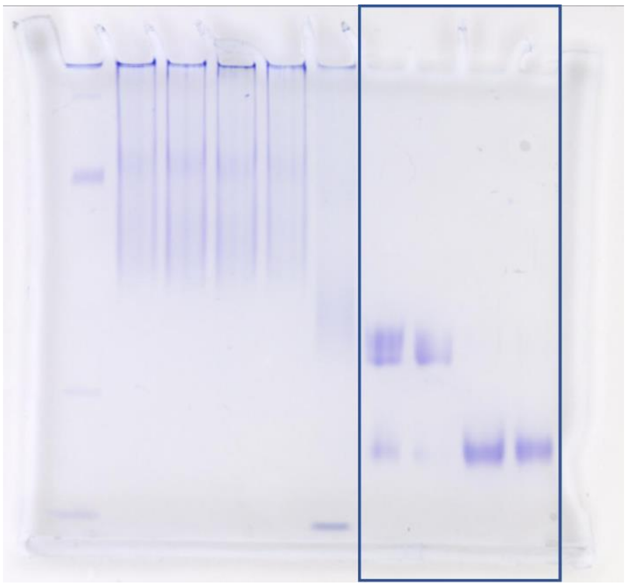

Supplementary Fig. 13 Uncropped native PAGE gel of Figure 2d. The lanes shown in Figure 2d are framed. The other lanes contain samples that were analysed in conditions not relevant to the present study.

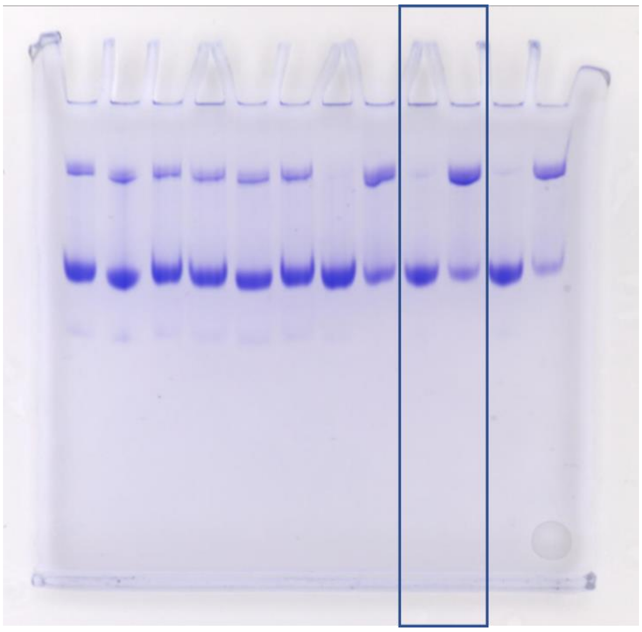

Supplementary Fig. 14 Uncropped native PAGE gel of Figure 3a. The lanes shown in Figure 3a are framed. The other lanes contain samples that were analysed in conditions not relevant to the present study.

Shape of the replot	Independent	Hyperbolic increase	Hyperbolic decrease	Linear increase
**Replot**				
$k_{cat\ app}$ vs. [X]	A	B	C	Does not exist
$1/k_{cat\ app}$ vs. [X]	D	E	F	G
$K_{m\ app}$ vs. [X]	H	I	J	K
$(k_{cat}/K_m)_{app}$ vs. [X]	L	M	N	Does not exist
$(K_m/k_{cat})_{app}$ vs. [X]	O	P	Q	R

Supplementary Table 1 Labelling of apparent kinetic parameters dependencies on inhibitor concentration. [X] refers to inhibitor (or activator) concentration. For each parameter, the shape of the dependency is associated with a particular letter. The unique combination of letters given by the shape of the replots allows to identify the associated kinetic mechanism of enzyme inhibition (or activation), as thoroughly explained in the website https://www.enzyme-modifier.ch/. An illustrated version of this Table can be found on this website.

Supplementary Table 2 Primers used for PCR amplification of pAVA0421-*Mt*SerB2 to synthesize variants *Mt*SerB2 ΔACT1 and *Mt*SerB2 Q92E (5'→3')

*Mt*SerB2 ΔACT1 forward	TCC TGG TTC GTC CAC CCA CAC CAT TTT CG
*Mt*SerB2 ΔACT1 reverse	TGT GGG TGG ACG AAC CAG GAC CCT GGG T
*Mt*SerB2 Q92E forward	ATT CGG GAA CCG TCC ACC CAC ACC ATT TTC GTG CTG
*Mt*SerB2 Q92E reverse	GGA CGG TTC CCG AAT GAT TGG CAG ATC GTC GCT GC

Supplementary Table 3 Accession codes and sequence parameters of mycobacterial PSPs studied in our work.

Enzyme	SSGCID ref. id	UniProt	Length*	Molar mass (Da)*	pI*	Ext. coef. ($M^{-1}\ cm^{-1}$)*
*Mt*SerB2	MytuD.01155.a	O53289	413	43,357	4.78	11460
*Mm*SerB2	MymaA.01155.a	B2HHH0	416	43,840	4.67	11460
*Ma*SerB	MyavA.01155.a**	A0QJI1**	416	43,907	4.77	7450

*These parameters were calculated using ProtParam tool (https://web.expasy.org/protparam/) on the basis of the exact sequences of the characterized constructs (including the N-term GPGS sequence remaining after HRV3C protease cleavage). pI: isoelectric point. The exact sequences were confirmed by plasmid sequencing.
** Mismatches were observed with the UniProt reference indicated by SSGCID: V inserted between M1 and N2 + mutations G31R and G152E.
