# Supplementary material for: A morpheein equilibrium regulates catalysis in phosphoserine phosphatase SerB2 from Mycobacterium tuberculosis
Source: Commun Biol. 2023 Oct 10;6:1024. doi: 10.1038/s42003-023-05402-z (PMC10564941; doi:10.1038/s42003-023-05402-z)
Supplement: Supplementary file 3 — reporting summary [file 42003_2023_5402_MOESM3_ESM.pdf]

nature portfolio

Corresponding author(s): WOUTERS Johan

Last updated by author(s): Sep 11, 2023

Reporting Summary

Nature Portfolio wishes to improve the reproducibility of the work that we publish. This form provides structure for consistency and transparency in reporting. For further information on Nature Portfolio policies, see our Editorial Policies and the Editorial Policy Checklist.

Statistics

For all statistical analyses, confirm that the following items are present in the figure legend, table legend, main text, or Methods section.

n/a	Confirmed	
☐	☒	The exact sample size (*n*) for each experimental group/condition, given as a discrete number and unit of measurement
☐	☒	A statement on whether measurements were taken from distinct samples or whether the same sample was measured repeatedly
☒	☐	The statistical test(s) used AND whether they are one- or two-sided<br>*Only common tests should be described solely by name; describe more complex techniques in the Methods section.*
☒	☐	A description of all covariates tested
☒	☐	A description of any assumptions or corrections, such as tests of normality and adjustment for multiple comparisons
☐	☒	A full description of the statistical parameters including central tendency (e.g. means) or other basic estimates (e.g. regression coefficient) AND variation (e.g. standard deviation) or associated estimates of uncertainty (e.g. confidence intervals)
☒	☐	For null hypothesis testing, the test statistic (e.g. *F*, *t*, *r*) with confidence intervals, effect sizes, degrees of freedom and *P* value noted<br>*Give P values as exact values whenever suitable.*
☒	☐	For Bayesian analysis, information on the choice of priors and Markov chain Monte Carlo settings
☒	☐	For hierarchical and complex designs, identification of the appropriate level for tests and full reporting of outcomes
☒	☐	Estimates of effect sizes (e.g. Cohen's *d*, Pearson's *r*), indicating how they were calculated

Our web collection on statistics for biologists contains articles on many of the points above.

Software and code

Policy information about availability of computer code

Data collection	Chromatography and SEC-MALS data were collected using Unicorn or Bio-SEC (Agilent) softwares, MP data were collected using DiscoverMP software version 2022 R1, SEC-SAXS data were collected at SOLEIL Synchrotron (Saint-Aubin, France) on the SWING beamline.
Data analysis	MP data were analysed using DiscoverMP software v 2022 R1 (Refeyn Ltd), SEC-SAXS data were analysed using Foxtrot software (courtesy of SWING beamline), BioXTAS RAW 2.1.1 and CRYSOL in PRIMUS/qt ATSAS 3.0.4 software, multiple sequence alignement was performed using PRALINE online tool (https://www.ibi.vu.nl/programs/pralinewww/), homology modeling was undertaken using SWISS-MODEL, 3D visualisation and modification of PDB files was performed using PyMOL (2006 DeLano Scientific LLC) or Maestro 11.9.011 (Schrödinger), MD simulation was run using GROMACS 2020, molecular models were generated using M-ZDOCK, ClusPro, GalaxyTongDock and CORAL on the online version of ATSAS, solution structures were refined against exp data using DADIMODO, enzyme kinetics data were analysed using GraphPadPrism 5 (GraphPad Software, La Jolla California USA).

For manuscripts utilizing custom algorithms or software that are central to the research but not yet described in published literature, software must be made available to editors and reviewers. We strongly encourage code deposition in a community repository (e.g. GitHub). See the Nature Portfolio guidelines for submitting code & software for further information.

Data

Policy information about availability of data

All manuscripts must include a data availability statement. This statement should provide the following information, where applicable:

- Accession codes, unique identifiers, or web links for publicly available datasets
- A description of any restrictions on data availability
- For clinical datasets or third party data, please ensure that the statement adheres to our policy

The SAXS data were deposited to SASBDB with accession code SASDRS4 for MtSerB2 dimer, SASDRT4 for MtSerB2 tetramer, SASDRU4 for MtSerB2 trimer, SASDRV4 for MmSerB2 dimer, and SASDRW4 for MaSerB dimer. Uncropped native PAGE gels are shown in Supplementary Figures 13 and 14. Numerical source data for enzyme kinetics graphs and MALS analyses, as well as MD simulation input files, and initial and final coordinates file, are publicly available on FigShare repository: https://doi.org/10.6084/ m9.figshare.24116571. All other data are available from the corresponding author on reasonable request.

Research involving human participants, their data, or biological material

Policy information about studies with human participants or human data. See also policy information about sex, gender (identity/presentation), and sexual orientation and race, ethnicity and racism.

Reporting on sex and gender	n/a
Reporting on race, ethnicity, or other socially relevant groupings	n/a
Population characteristics	n/a
Recruitment	n/a
Ethics oversight	n/a

Note that full information on the approval of the study protocol must also be provided in the manuscript.

Field-specific reporting

Please select the one below that is the best fit for your research. If you are not sure, read the appropriate sections before making your selection.

☒ Life sciences ☐ Behavioural & social sciences ☐ Ecological, evolutionary & environmental sciences

For a reference copy of the document with all sections, see nature.com/documents/nr-reporting-summary-flat.pdf

Life sciences study design

All studies must disclose on these points even when the disclosure is negative.

Sample size	Enzyme kinetics data are reported as means ± s.d. of three independent enzymatic reactions per condition of substrate concentration, and condition of L-Ser concentration in inhibition kinetics measurements.
Data exclusions	A few individual data points (absorbance) were excluded when the s.d. exceeded a treshold value of 0.1 for substrate concentrations below 1mM and 0.25 for substrate concentrations above 1mM.
Replication	Reproducibility was monitored through s.d. calculation. The measurements were reproductible.
Randomization	All replicate reactions were run with a volume of enzyme solution coming from the same sample (same tube).
Blinding	Blinding is not relevant in enzyme kinetics studies.

Reporting for specific materials, systems and methods

We require information from authors about some types of materials, experimental systems and methods used in many studies. Here, indicate whether each material, system or method listed is relevant to your study. If you are not sure if a list item applies to your research, read the appropriate section before selecting a response.

Materials & experimental systems

n/a	Involved in the study
☒	☐ Antibodies
☒	☐ Eukaryotic cell lines
☒	☐ Palaeontology and archaeology
☒	☐ Animals and other organisms
☒	☐ Clinical data
☒	☐ Dual use research of concern
☒	☐ Plants

Methods

n/a	Involved in the study
☒	☐ ChIP-seq
☒	☐ Flow cytometry
☒	☐ MRI-based neuroimaging
